# Murine Myeloid Dendritic Cells That Phagocytose Apoptotic T Cells Inhibit the Immune Response via NO

**DOI:** 10.1371/journal.pone.0049378

**Published:** 2012-11-15

**Authors:** Kaili Zhong, Wengang Song, Qian Wang, Chao Wang, Xi Liu, Dongwei Chen, Zhongli Zhu, Yiqing Wu, Weijing Zhang, Minghui Zhang

**Affiliations:** 1 Department of Lymphoma, Affiliated Hospital of Academy of Military Medical Sciences, Beijing, People’s Republic of China; 2 Department of Immunology, Taishan Medical College, Tai’an, Shandong Province, People’s Republic of China; 3 Institute of Immunology, School of Medicine, Tsinghua University, Beijing, People’s Republic of China; University Hospital Freiburg, Germany

## Abstract

The contraction phase of antigen-specific immune responses involves the apoptotic loss of numerous activated lymphocytes. While apoptotic cells are known to induce immune suppression, the mechanisms involved therein are still ambiguous. Some reports have speculated that macrophages can induce regulatory T cells (Tregs) after engulfing apoptotic cells. In this study, we showed that dendritic cells (DCs) that phagocytose apoptotic T cells acquire inhibitory function (named DCapos) toward CD4^+^ and CD8^+^ T cells. These inhibitory DCs could not induce the generation of Tregs, but they were found to directly inhibit mDCs that initiate CD4^+^ and CD8^+^ T cell proliferation both in vitro and in vivo. Soluble factors including NO play a role in the DCapos-induced suppression of CD4^+^ and CD8^+^ T cell proliferation. Further results showed that STAT3 phosphorylation and inducible nitric oxide synthase (iNOS) generation were enhanced when DCs were co-cultured with apoptotic cells. Both iNOS transcription and NO secretion were inhibited in the presence of the specific p-STAT3 inhibitor JSI-124. All the data indicated that apoptotic cells could turn DCs to inhibitory DCs, which might play important roles in the suppression of immune responses. STAT3 activation and the consequent release of NO are responsible for the inhibitory functions of DCapos.

## Introduction

Activation-induced cell death (AICD) of T cells is a key mechanism for downregulating the immune response. For apoptotic cell clearance, antigen-presenting cells (APCs) phagocytose apoptotic T cells to maintain homeostasis of the immune system. These APCs that have phagocytosed apoptotic T cells can also induce immune tolerance, which is considered to be due to the subsequently induced Treg’s effect [1], [2]. However, it is yet unknown whether other mechanism(s) are involved in apoptosis-induced immune tolerance.

DCs are pivotal professional APCs with important functions both in initiating immune response and in maintaining immune tolerance. It has been demonstrated that a distinct group of DCs with suppressive function emerge after exposure to certain cytokines or when the DCs re-differentiation in different stromal microenvironments [3], [4], [5]. The cytokines or factors produced by DCs, such as transforming growth factor-beta (TGF-beta), IL-10, indoleamine 2,3-dioxygenase (IDO), and NO, may play an important role in the regulation of immune responses [1], [6], [7], [8], [9]. In our previous studies, we demonstrated that NO production is a significant characteristic of regulatory DCs derived from mature DCs co-cultured with splenic or hepatic stroma [3], [10] and that this NO exerts direct, rapid and potent inhibitory effects on T cell proliferation.

Our preliminary data showed that DCs also produce NO after phagocytosing apoptotic T cells, which are similar to regulatory DCs derived from mature DCs co-cultured with stoma, suggesting that after phagocytosing apoptotic T cells, DCs may directly inhibit the immune response. Here, we evaluated the regulatory function of DCs after phagocytosing apoptotic T cells and investigated the relationship between the phagocytosis of apoptotic T cells and NO production in DC.

## Materials and Methods

### Mice and Cell Lines

Six-week-old C57BL/6 (H-2Kb) mice and BALB/c (H-2Kd) mice were purchased from Vitariver (Beijing, China). OVA323–339-specific TCR-transgenic mice DO11.10 (H-2K^d^), OVA257–264-specific TCR-transgenic mice OT-1, Foxp3^EGFP^ mice (H-2K^d^), and EGFP-transgenic mice C57BL/6-Tg (ACTb-EGFP) (H-2K^b^) were obtained from the Jackson Laboratory (Bar Harbor, ME). F1 mice were prepared by crossing C57BL/6 mice with DO11.10 mice (C57BL/6×DO11.10). Another set of F1 mice were prepared by crossing Foxp3^EGFP^ with DO11.10 mice (Foxp3^EGFP^×DO11.10). All the mice were maintained under specific pathogen-free conditions and used at 6–8 weeks of age. All experimental manipulations were undertaken in accordance with the National Institute of Health Guide for the Care and Use of Laboratory Animals, with the approval of the Tsinghua University Animal Care and Use Committee, Beijing.

### Reagents

CFSE, STAT3 inhibitor JSI-124 (cucurbitacin I), 1,4-phenylene-bis(1,2-ethanediyl)bis-isothiourea dihydrobromide (1,4-PBIT), and the NO donor NOC-18 were all purchased from Sigma-Aldrich (St. Louis, MO). Magnetic beads conjugated with mAbs to CD4, CD11b, and CD11c were purchased from Miltenyi Biotec (Bergisch Gladbach, Germany). Fluorescein-conjugated mAbs to CD4, CD8a, CD11b, CD11c, CD40, CD45.1, CD80, CD86, Ia, KJ1-26, Vβ5.1/5.2, and isotype control mAbs were purchased from BD Pharmingen (San Diego, CA). Specific Abs against STAT3 (Cell Signaling Technology, Beverly, MA), phosphor-STAT3 (Tyr705) (Cell Signaling Technology), iNOS (Cell Signaling Technology), beta-actin (Santa Cruz Biotechnology) were used.

### Preparation of Activated and Apoptotic CD4^+^ T Cells

Splenic CD4^+^ T cells from DO11.10×C57BL/6 F1 hybrid mice were obtained by magnetic cell sorting and then co-cultured in 24-well plates with mDCs (DCs that were stimulated by LPS 1 ng/ml for more than 24 hours) for 5 days at a ratio of 1∶10 in the presence of OVA323–339. The CD4^+^ T cells were sufficiently activated and used as activated CD4^+^ T cells. Thereafter the CD4^+^ T cells were obtained by negative selection with CD11b magnetic microbeads and subsequent cultured without mDCs for another 2 days to induce apoptosis of more than 70% of the CD4^+^ T cells. The CD4^+^ T cells were stained by Hoechst 33342, and then centrifuged at 100 *g* to discard intact cells and obtain the apoptotic cells. The supernatants were centrifuged again at 350 *g*, and annexin-V and propidium iodide (PI) staining with flow cytometry revealed that the resultant sediment contained more than 95% apoptotic cells and less than 5% necrotic lymphocytes, which were used as apoptotic T cells.

### Generation of DCs and DCapos

Bone marrow-derived DCs were prepared from male or female mice in the presence of GM-CSF and IL-4 as described previously [3]. Floating cells were collected on day 7, and CD11c^+^ DCs were purified by CD11c magnetic microbeads [3] and used as DCs in the study, the purity of DCs should reach above 95%. DCs were stimulated by LPS for more than 24 hours and used as mDCs to stimulate T cells. And the DCs were co-cultured with apoptotic T cells at a ratio of 1∶5 (DCs:apoptotic cells) for more than 3 days or for the indicated times. Then the CD11b^+^Hoechst^+^ cells were sorted by flow cytometry and used as DCapos, the purity of DCs should reach above 95% [1]. In the experiment of T cells proliferation *in vitro* and *in vivo*, DCapos were stimulated by LPS 1 ng/ml for 24 h before coculture with T cells or transfer into mice. In some experiments, DCapos (2×10^6^ cells per well) were treated with JSI-124 for 1 h before apoptotic cell treatment or LPS stimulation. The supernatants were then collected for NO assay.

### Analysis of Phagocytosis Ability and Phenotypes of DCs

DCs and DCapos were incubated with Alexa 488-conjugated OVA at a final concentration of 100 µg/ml, and the geometric mean fluorescence was detected using FACSAria [3]. For phenotype analysis, DCs and DCapos were blocked using rat serum before they were stained with fluorescein-conjugated mAbs and detected by FACSAria.

### In vitro Proliferation and Activation Assay

As described previously [3], splenic CD4^+^ T cells were positively selected from DO11.10×C57BL/6 F1 hybrid mice by using magnetic-activated cell sorting for use as antigen-specific responders. The CD4^+^ T cells were then co-cultured for 5 days with live mDCs or DCapos in 96-well plates (mDCs:T cells, DCapos:T cells, 1∶10) in the presence of OVA323–339 (2×10^5^ T cells in 250 µl per well). The CD8^+^ T cells were also assayed according to the above mentioned method. Splenic CD8^+^ T cells from OT1 mice were positively selected by magnetic activated cell sorting, and co-cultured for 3 days with live mDCs or DCapos in 96-well plates (mDCs:T cells, DCapos:T cells, 1∶10) in the presence of OVA257–264 (2×10^5 ^T cells in 250 µl per well). The supernatants were collected for the cytokine assay. The cells were double stained with CD4-APC or CD8a-APC and 7-AAD, and the number of CD4^+^/CD8^+^ 7-AAD^–^ cells were analyzed using FACSAria. To detect the activation of T cells, CD25-APC or CD69-APC combined with CD4-FITC, KJ1-26-PE were used. To investigate the development of Tregs, splenic CD4^+^ T cells were prepared from Foxp3^EGFP^×DO11.10 F1 mice.

### In vivo Transfer of Differentiated DCs

CFSE-labeled OVA323–339-specific TCR-transgenic splenic CD4 T cells (5×10^6^ cells) were freshly isolated from DO11.10×C57BL/6 F1 hybrid mice, with OVA323–339-loaded mDCs and/or DCapos injected intraperitoneally into each F1 (BALB/c×C57BL/6) hybrid mouse. After 4 days, mononuclear cells from peripheral blood were prepared and double stained with CD4-APC and KJ1-26-PE. The CD8 T cells were transferred in the same way. CFSE-labeled OVA257–264-specific TCR-transgenic splenic CD8 T cells (1×10^6^ cells) were freshly isolated from OT1 mice, with OVA257–264-loaded mDCs and/or DCapos injected intraperitoneally into each C57BL/6 mouse. After 3 days, mononuclear cells from the peripheral blood were prepared and double stained with CD8-APC and Vβ5.1/5.2-PE. To investigate the development of Tregs in vivo, apoptotic cells from BALB/c mice were prepared and injected intraperitoneally into Foxp3^EGFP^ or (Foxp3^EGFP^×DO11.10) F1 mice.

### Assay for Cytokines and NO

We collected supernatants for detection of cytokines. Cytokines were detected on a commercial ELISA kit (Bender Medsystems) according to the manufacturer’s instructions. NO production was determined by measuring the nitrite concentration by Griess assay, as described previously [10].

### RT-PCR

Total cellular RNA from DCs or DCapos was isolated by using TRIzol reagent (Invitrogen). The RNA was reverse transcribed using a TransScript II First-Strand cDNA Synthesis SuperMix (TransGen Biotech, Beijing, China). The RT-PCR was carried out using the following mouse-specific primer pairs: iNOS (forward: 5′-ACCACCCTCCTCGTTC-3′, reverse: 5′-GCCTATCCGTCTCGTC-3′); TGF-beta1 (forward: 5′-ATACAGGGCTTTCGATTCAGC-3′, reverse: 5′-GTCCAGGCTCCAAATATAGG-3′); IL-10 (forward: 5′-ACCACCCTCCTCGTTC-3′, reverse, 5′-GCCTATCCGTCTCGTC-3′).

### Western Blotting

Western blotting was carried out as described [11]. Apoptotic cells were added to untreated control DC cultures immediately before preparation of the lysates, in order to exclude AC-derived proteins occurring after AC pretreatment of DCs from interfering with the protein expression analysis. Membranes were probed with Abs specific for STAT3, phosphor-STAT3 (Tyr705), iNOS, and beta-actin. Binding of secondary horseradish peroxidase (HRP)-labeled goat α-rabbit, or goat α-mouse Abs (Santa Cruz Biotechnology) was analyzed using an ECL kit (GE Healthcare, Amersham).

### Statistical Analyses

Data were obtained from 3 independent experiments. All statistical analyses were performed with ANOVA using SPSS version 11.0. A value of p < 0.05 was considered statistically significant.

## Results

### Apoptotic T Cells Induce Functional and Phenotype Changes in DCs

**Figure 1:**
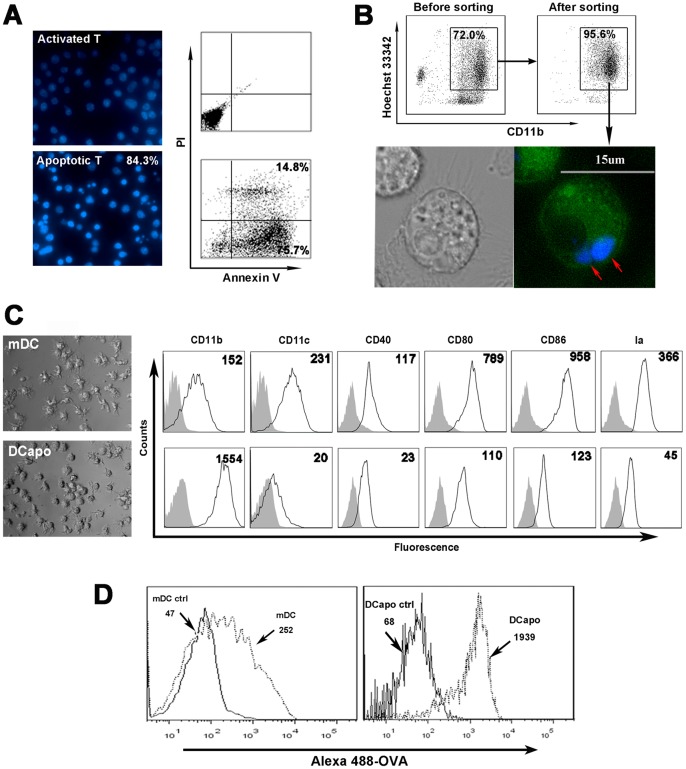
Morphology, phenotype, and endocytic ability of mDCs and DCapos. ( A) Apoptosis of CD4^+^ T cells. Left panel, top left (×400): CD4^+^ T cells stained by Hoechst 33342 after activation by mDCs and OVA_323–339_ for 2 days; bottom left (×400): after activation by mDCs and OVA_323–339_ for 5 days, CD4^+^ T cells were cultured for another 2 days in the absence of mDCs and then stained by Hoechst 33342. Numbers showed the percentage of apoptotic cells in high power field. Upper right panel, control cells. Lower right panel, apoptotic CD4^+^ T cells were assessed by flow cytometry with Annexin V and PI. (B) Purification of DCapos. Top panel, DCs were co-cultured with apoptotic CD4^+^ T cells prelabeled with Hoechst 33342. Then the CD11b^+^Hoechst^+^ cells were sorted by flow cytometry. Numbers showed the purity of the sorted cells. Bottom panel, DCs incubated with apoptotic CD4^+^ T cells prelabeled with Hoechst 33342 (blue with red arrows) and incubated for 2 hours. The DCs were then stained with CD11b-FITC (green) and observed under a light microscope (left) and a confocal immunofluorescence microscope (right). Bar represent 15 µm. (C) Morphologies and phenotypes of mDCs and DCapos. Left panel, left top (×400), the morphology of mDCs; left bottom (×400), the morphology of DCapos. Right panel, different groups of DCs were stained with fluorescence-conjugated anti-CD11b, anti-CD11c, anti-CD80, anti-CD86, anti-CD40, and anti-Ia mAbs for flow cytometric analysis. Numbers represented the mean fluorescence intensity. (D) Endocytic abilities of mDCs and DCapos were assessed by flow cytometry of Alexa 488-OVA uptake. Numbers in the histograms indicate the geometric mean fluorescence of each DC population. Ctrl, control (cells incubated with Alexa 488-OVA at 4°C). Data represent results of 1 of the 3 experiments performed to yield similar results.

Naïve ovalbumin (OVA)-specific TCR transgenic CD4^+^ T cells can be activated by DCs in the presence of OVA peptide (OVA323–339). Approximately 70–80% of activated T cells undergo apoptosis when cultured for an additional 2 days without DCs; which can be verified by the morphological changes observed in the T cells (Fig. 1A, left) or by annexin-V/PI staining (Fig. 1A, right). Apoptotic T cells were harvested by centrifugation and co-cultured with bone marrow-derived mature DCs (BMDCs) for another 3 days,and then the CD11b^+^Hoechst^+^ cells were sorted (Fig. 1B, top). As shown in (Fig. 1B, bottom), the apoptotic T cells rapidly engulfed by mDCs after they were co-cultured with DCs for 2 h. Thus, these mDC which engulfed apoptotic T cells were termed DCapos. To characterize these DCapos, we explored both the morphological and phenotypic changes in mDCs after they were exposed to apoptotic T cells for 3 days. As shown in Fig. 1C, similar to stroma-educated regulatory DCs, DCapos are also characterized by upregulated CD11b expression but downregulated CD11c, Ia, CD80, CD86, and CD40 expressions [3], [12], [13]. After the phagocytosis of apoptotic cells, mature DCs adhered to the flask to a greater extent and showed more potent phagocytic ability (Fig. 1D); nevertheless, LPS treatment could not induce maturation of these DCs. The data suggested that phagocytosis of apoptotic cells can induce changes in the phenotype and function of DCs.

### DCapos Activated T Cells but Inhibited their Proliferation in vitro and in vivo

**Figure 2:**
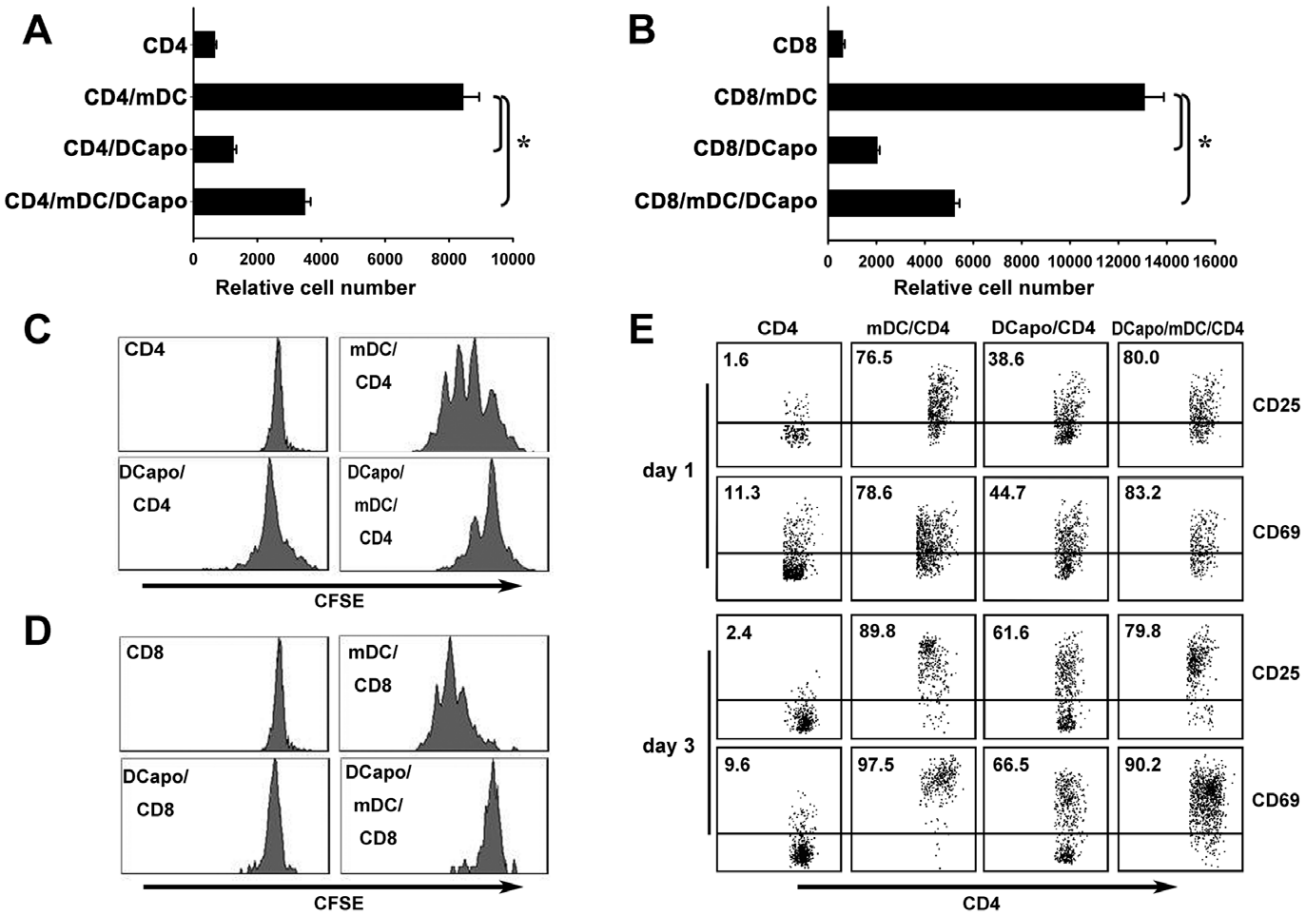
DCapos activated T cells but inhibited their proliferation. For CD4^+^ T cells, purified CD4^+^ T cells from DO11.10×C57BL/6 F1 hybrid mice (2×10^5^ cells) stained with CFSE were co-cultured in vitro with OVA_323–339_-loaded mDCs (2×10^4^ cells) in the presence or absence of DCapos (2×10^4^ cells) for 4 days. For CD8^+^ T cells, purified OT1-CD8^+^ T cells (2×10^5^ cells) stained with CFSE were co-cultured in vitro with OVA_257–264_-loaded mDCs (2×10^4^ cells) in the presence or absence of DCapos (2×10^4^ cells) for 3 days. (A) The cells were collected at 4 days, and both CD4- and KJ1-26-positive cells were counted using flow cytometry. (B) The cells were collected at 3 days, and both CD8- and Vβ5.1/5.2-positive cells were counted using flow cytometry. Histograms showed the mean ± SEM of triplicate wells counted using flow cytometry, and results represent 3 separate experiments. *p < 0.05. (C, D) The CFSE dilutions of CD4^+^ and CD8^+^ T cells were analyzed using flow cytometry. (E) Cells from different CD4^+^ T cell groups were collected and stained with anti-CD4, anti-CD25, and anti-CD69 mAbs for flow cytometric analysis. Data represent results of 1 of the 3 experiments performed to yield similar results.

Naïve TCR-transgenic CD4^+^ T cells obtained from DO11.10×C57BL/6 F1 mice (Fig. 2A, C) or TCR-transgenic CD8^+^ T cells from OT1 mice (Fig. 2B, D) were used as responders to evaluate the antigen-presenting ability of DCs and DCapos in the presence of the OVA peptides, namely, OVA323–339 and OVA257–264. T cell counting (Fig. 2A, B) and carboxyfluorescein diacetate succinimidyl ester (CFSE) division (Fig. 2C, D) were assayed by flow cytometry after co-culture for 5 days. The results revealed that mature DCs are capable of stimulating vigorous T cell proliferation, but the DCapos lost this ability even after LPS stimulation. When DCapos were added to a mature DC/T cell co-culture system, the proliferation of the T cells was potently inhibited. Though DCapos were able to inhibit T cell proliferation, most of the T cells co-cultured with DCapos were found to express activation-related surface markers such as CD25 and CD69, suggesting that DCapos still had the ability to activate T cells (Fig. 2E ).

### DCapos Inhibited T Cell Proliferation in vivo

**Figure 3:**
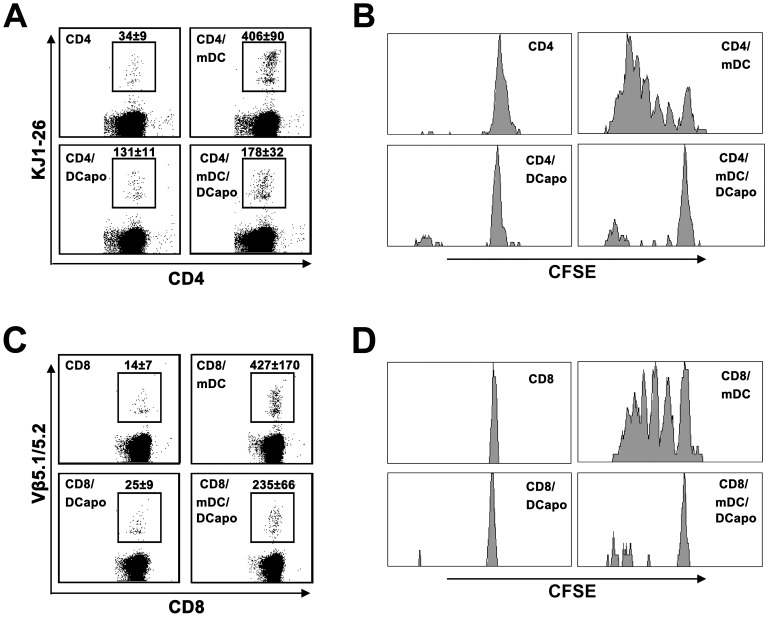
Inhibition of T cells proliferation by DCapos in vivo. ( A) CD4^+^ T cells (5×10^6^ cells) from DO11.10×C57BL/6 F1 hybrid mice stained with CFSE, OVA_323–339_-loaded mDCs (1×10^6^ cells) with or without DCapos (1×10^6^ cells) were transferred together into the peritoneal cavity of normal mice for 4 days. Mononuclear cells from the blood were separated and stained for flow cytometric analysis of the frequency of both CD4- and KJ1-26-positive T cells. (B) The CFSE dilutions of CD4^+^KJ1-26^+^ T cells were analyzed using flow cytometry. (C) OT1 CD8^+^ T cells (1×10^6^ cells), OVA_257–264_-loaded mDCs (3×10^5^ cells) with or without DCapos (3×10^5^ cells) were transferred together into the peritoneal cavity of normal mice for 3 days. Then, the mononuclear cells were separated from the blood and stained for flow cytometric analysis of the frequency of both CD8- and Vβ5.1/5.2-positive T cells. (D) The CFSE dilutions of CD8^+^ Vβ5.1/5.2^+^ T cells were analyzed by flow cytometry. Data are presented as mean ± SD of 5 mice, and results represent 1 of the 2 experiments with similar results.

To further confirm the inhibitory function of DCapos, we adoptively transferred naïve transgenic CD4^+^ and CD8^+^ T cells together with the corresponding peptide-loaded mDCs and DCapos into donor mice to test T cell proliferation. In our previous study, we obtained consistent results for the blood and spleen T cells. Therefore, the transferred CD4^+^ and CD8^+^ T cells in the blood can be recognized by the corresponding TCR-specific monoclonal antibodies. Direct cell counting (Fig. 3A, C) and CFSE division (B, D) assay revealed that mature DC-induced T cell proliferation was potently inhibited by DCapos in vivo (Fig. 3).

### NO Played a Critical Role in the Inhibitory Function of DCapos

**Figure 4:**
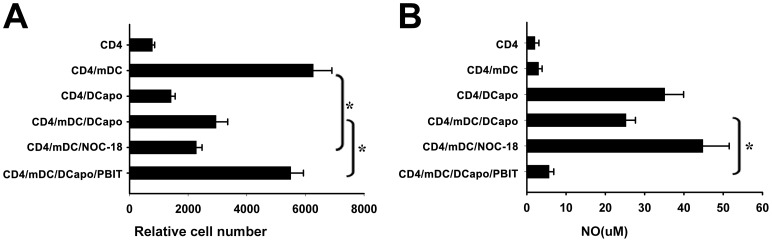
DCapos inhibited CD4^+^ T cell proliferation via NO. Purified DO11.10 CD4^+^ T cells (2×10^5^ cells) stained with CFSE were co-cultured with OVA_323–339_-loaded mDCs (2×10^4^ cells) in the presence or absence of DCapos (2×10^4^ cells) for 4 days in vitro. (A) The NO donor NOC-18 and the NOS inhibitor PBIT were added to the co-culture system to evaluate their effect on the inhibitory function of DCapos. (B) The supernatants of different groups were collected and the NO secretions were tested by Griess assay. *p < 0.05. Data are presented as mean ± SD of triplicate wells, and the results represent 3 separate experiments.

Our previous studies on stroma-educated regulatory DCs (DCregs) suggested that NO is an important soluble factor released by DCregs, and is involved in their immunosuppression [3], [12]. These results led us to question whether NO was involved in the inhibitory function of DCapos. Our study revealed that the selective NO synthase inhibitor 1,4-phenylene-bis(1,2-ethanediyl)bis-isothiourea dihydrobromide (1,4-PBIT, 10 µM) effectively reversed DCapos inhibition of CD4^+^ T cell proliferation stimulated by mDCs (Fig. 4A). However, we found that the addition of 5 µM NOC-18 to antigen-specific CD4^+^ T cell/mDC co-culture system significantly suppressed T cell proliferation (Fig. 4A). Then, the supernatant was collected from different groups and the NO concentration was measured by Griess assay. The results demonstrated that the NO concentration is associated with the immunosuppressive effects of DCapos (Fig. 4B).

### Foxp3^+^ Tregs was Not Involved in the Inhibitory Function of DCapos

**Figure 5:**
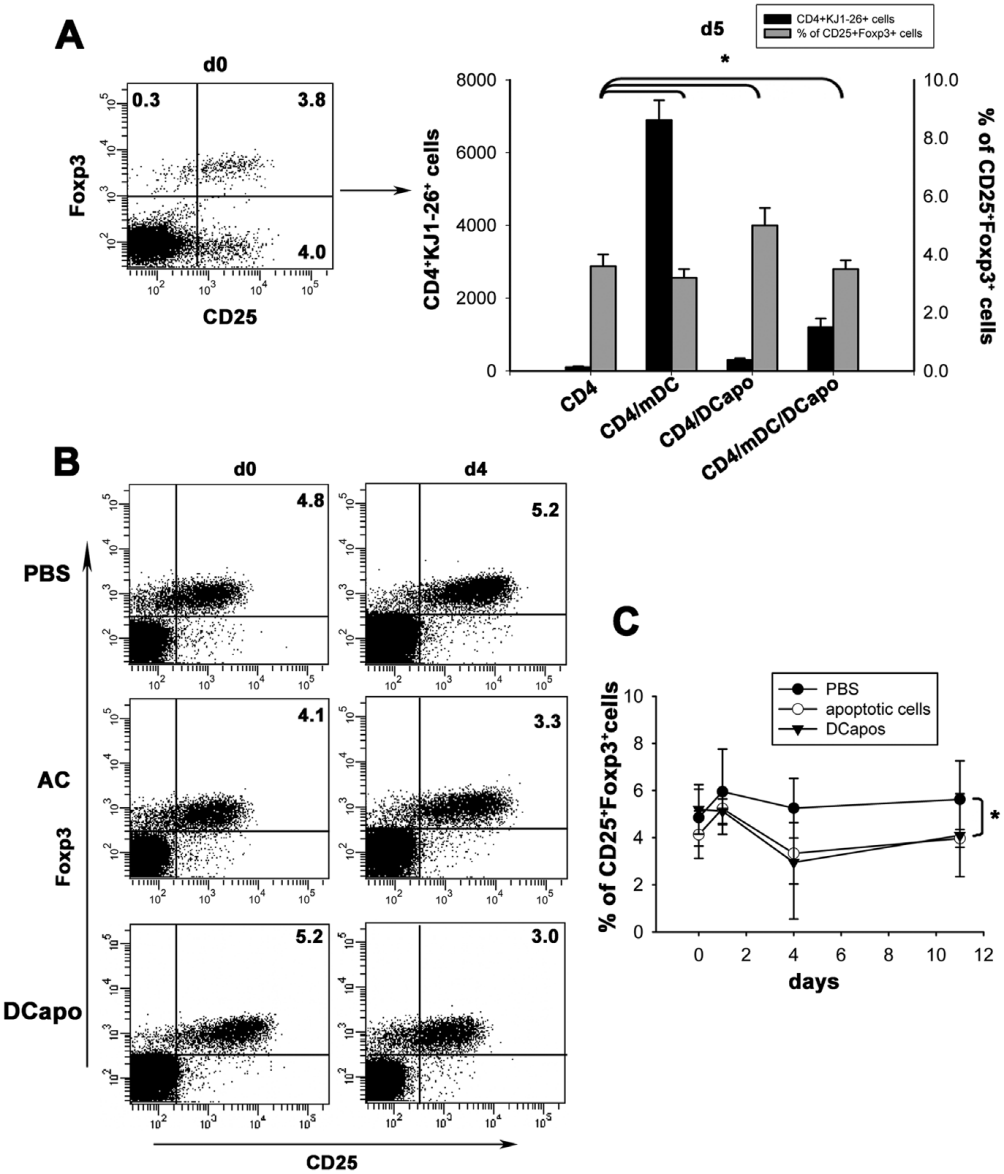
Foxp3^+^ Tregs were not involved in the inhibitory function of DCapos both in vitro and in vivo. (A) Left, DO11.10 CD4^+^ T cells (2×10^5^ cells) with Foxp3^EGFP^ were purified, and the CD4^+^CD25^+^Foxp3^+^ cells were measured in a FACS system (numbers indicate percentages). These cells were then co-cultured with OVA_323–339_-loaded mDCs (2×10^4^) in the presence or absence of DCapos (2×10^4^ cells) for 5 days in vitro. On the fifth day, the cells were harvested and the numbers of CD4- and KJ1-26-positive cells were determined by FACS analysis (black). The proportion of CD4^+^KJ1-26^+^CD25^+^Foxp3^+^ cells was determined by FACS (grey). Data are presented as mean ± SD of triplicate wells, and the results represent 2 separate experiments. (B, C) Apoptotic cells (AC), DCapos, or phosphate-buffered saline (PBS) was injected into the peritoneal cavity of Foxp3^EGFP^ mice (PBS, *n = *5, AC, *n = *8, DCapo, *n = *5). The CD4^+^CD25^+^Foxp3^+^ cells in the peripheral blood were assayed by FACS at days 0, 1, 4, and 11 (numbers indicate percentages). Data in b–c are representative of 3 separate experiments. *p > 0.05.

Tregs are known to play an indispensable role in maintaining immunological unresponsiveness to self-antigen and in suppressing excessive immune responses that are deleterious to the host. We then evaluated whether Tregs were involved in the DCapos-mediated suppression of the specific immune responses. We used hybrid mice co-expressing enhanced green fluorescent protein (EGFP) and the regulatory T cell-specific transcription factor Foxp3 on OVA323–339-specific TCR-transgenic CD4^+^ T cells. As compared to the CD4^+^ group, the percentage of Foxp3^+^CD25^+^ cells in the DCapo/CD4 group slightly increased but without statistically significant difference. Surprisingly, in the mDC/CD4 group, the Foxp3^+^CD25^+^ cells proliferated according to the total cell number. There was no evidence that DCapos induced the generation of Tregs (Fig. 5A). To further confirm the result *in vivo*, we adoptively transferred apoptotic T cells or DCapos into transgenic mice co-expressing EGFP and Foxp3. Foxp3^+^CD25^+^CD4^+^ cells were not involved in this process on day 4 or day 11 (Fig. 5B, C). The results indicated that Tregs could not be induced by apoptotic cells or DCapos.

### Cross-linking of Stat3 between DCapos and NO

**Figure 6:**
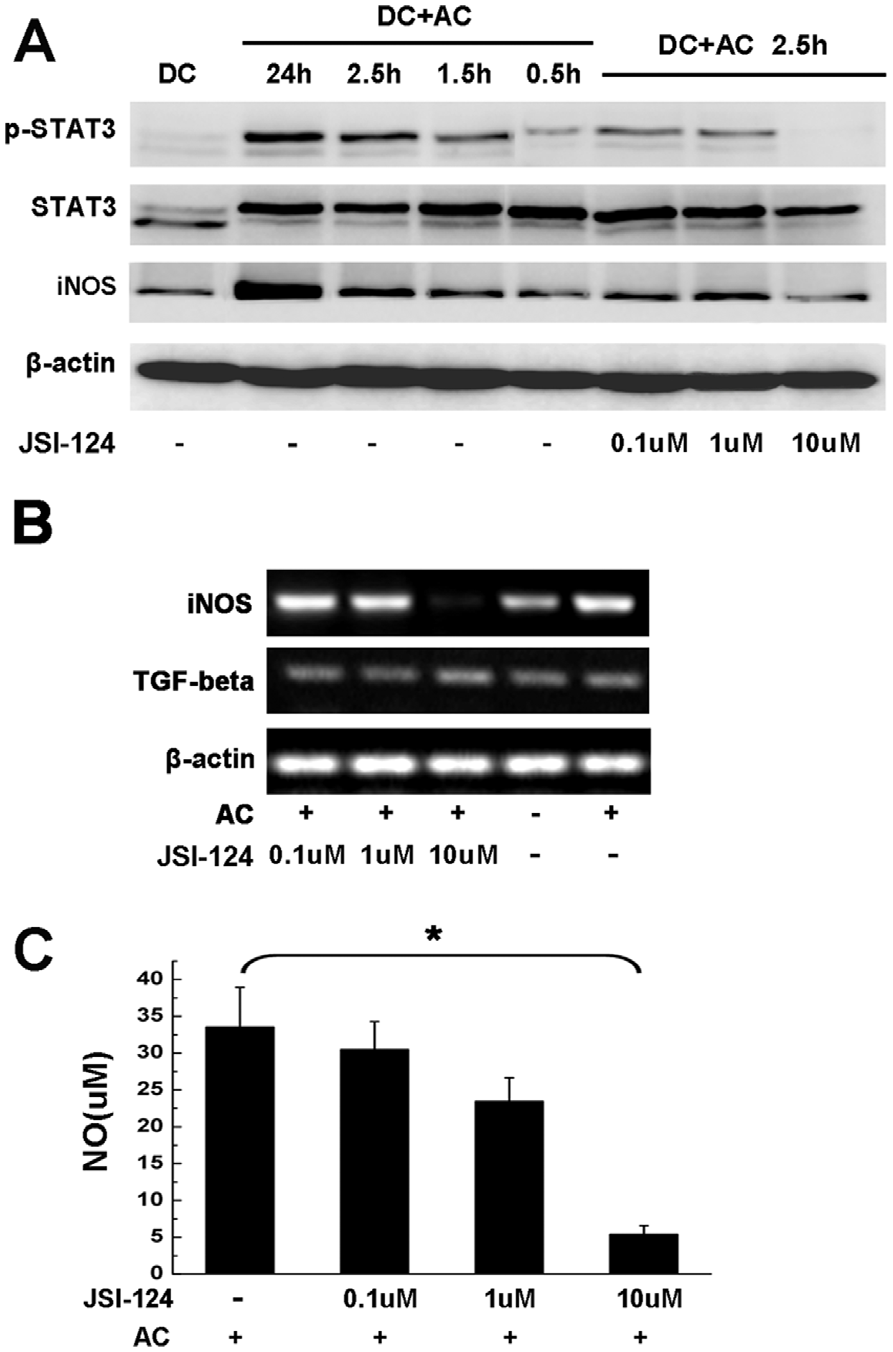
Cross-linking of STAT3 between DCapos and NO. (A) Apoptotic cells (AC) induced STAT3 activation in DCapos. DCs incubated with or without different concentrations of JSI-124 for 1 h were treated with or without ACs for the indicated times, then stimulated with 50 ng/ml LPS for 0.5 hours. The p-STAT3, STAT3, iNOS, and β-actin proteins were measured in whole-cell lysates by western blotting using the same membranes. (B) DCs were incubated with or without different doses of JSI-124 for 1 h, co-cultured with or without ACs for 2 h, then cultured for 24 hours before the cells were used for RT-PCR assay of mRNA expression of iNOS, TGF-β, and β-actin. Data represents 1 of 3 experiments with similar results. (C) The supernatants of different groups were collected and NO was analyzed by the Griess assay. *p < 0.05. Data represent the mean ± SEM of 3 separate experiments.

We further explored the mechanisms of AC-induced inhibition by DCapos. Recently, the tyrosine kinase Mer (MerTK) receptor was implicated in the blockade of NF-kappaB activation by apoptotic cells in DCs [14]. Since the intracellular domain of MerTK contains a binding motif (YPGV) for the STAT3 transcription factor, we used western blotting to investigate p-STAT3 activation in DCs co-cultured with apoptotic cells for indicated times. Our results revealed increased STAT3 phosphorylation in whole-cell lysates prepared from DCs after AC treatment, which was associated with treatment time. Further, the phosphorylation could be completely inhibited by the specific STAT3 phosphorylation inhibitor JSI-124 (10 µm). Moreover, it is noteworthy that the level of the STAT3 protein remained unaltered (Fig. 6A).

Previous data have demonstrated that DCapos exert inhibitory functions via NO. Since STAT3 signaling was involved in blocking DC activation [15], [16], [17], [18], we wondered whether or not any relation existed between STAT3 and NO production. As shown in Fig. 6A, iNOS synthesis was found to increase with time in AC-treated DCs; further, this increase could be inhibited by JSI-124. To confirm this result, we used RT-PCR to investigate the mRNA transcription of iNOS. After treatment with apoptotic cells, iNOS mRNA transcription increased and could be suppressed by 10 µm JSI-124 while the mRNA transcription of TGF-beta did not change (Fig. 6B). Similar results were observed in different DCapos groups, wherein NO secretion was similarly inhibited by 10 µm JSI-124 (Fig. 6C). Thus, AC could induce STAT3 phosphorylation in DCs, leading to the induction of NO in DCs and the ensuing inhibitory function of DCs.

## Discussion

Our previous study had demonstrated that mature DCs in stromal microenvironment could re-differentiate into DCregs after antigen presentation, but not undergo apoptosis [3]. It is also known that many lymphocytes undergo AICD during immune response in lymph organs. Moreover, apoptotic cells, but not necrotic cells, have been reported to trigger immunosuppression by phagocytosis [1], [7], [8], [19], [20], [21], [22], [23]. However, the intrinsic mechanisms for this phenomenon remain unclear, although some researchers have revealed that macrophages can induce the generation of Tregs after phagocytosis. Considering that DCs can release considerable amounts of NO after phagocytosing apoptotic T cells, the question whether or not these cells directly inhibit the immune response should be explored.

In this study, we constructed a model in which most lymphocytes underwent AICD after the stimulation of mature DCs. Some molecules displayed on the surface of apoptotic cells (AC), particularly phosphatidylserine, are reportedly recognized as “eat-me” signals by DCs [24]. We found that after co-culture of DCs with apoptotic cells, DCs could phagocytose the apoptotic cells or apoptotic bodies; these DCapos were different from mature DCs in terms of their phenotype and phagocytosis ability. Although the phenotype of DCapos was similar to that of macrophages, they were functionally different from macrophages but similar to the DCregs reported previously [3]. DCapos were generated from purified CD11c+ dendritic cells, but after engulfing apoptotic T cells, an enhanced CD11b expression were observed obviously. CD11b upregulation is a very important characteristic of mDC derived regulatory DCs, which have demonstrated in our previous research [3], [5], [10], [13]. Once the DCs turned to regulatory DCs, the CD11c would be downregulated and CD11b would be upregulated.

DCapos could significantly inhibit the proliferation of CD4^+^ or CD8^+^ T cells stimulated by mature DCs, indicating that DCapos are inhibitory cells. Various immunosuppressive factors have been proposed to be responsible for the inhibitory functions of DCs, such as TGF-beta, IL-10, IL-13, and NO [25]. In our study, we illustrated the critical role of NO in the inhibitory function of DCapos, which could be reversed by the NO inhibitor. We found that the NO levels in the groups containing DCapos were considerably higher than corresponding levels in other groups. It is known that NO is not stable and has a short range of action; therefore, it is surprising to note such strong immunosuppressive effects exerted by NO. We demonstrated that DCapos could activate naïve CD4^+^ T cells, and it has been suggested that DCapos might be more activated by the cytokines secreted by T cells and produce more amounts of NO [3].

Some studies have shown that DCs that phagocytose apoptotic cells could induce the differentiation of naive T cells into Tregs, especially into Foxp3^+^ Tregs, which contribute to the suppression of immune responses and immune tolerance [1], [2], [19], [20]. In our research system, no evidence indicated that Tregs participated in the inhibitory function of DCapos. There was no significant change in the percentage of Tregs in the peripheral blood of Foxp3-GFP transgenic mouse. It was reported that the CD4^+^CD25^+^Foxp3^+^ cells would proliferate or generate if allogeneic splenocytes were phagocytosed by DCs [2], [26]. Apoptotic DCs but not splenocytes or thymocytes have been reported to have the ability to convert immature DCs into tolerogenic DCs that induce differentiation of Foxp3^+^ Tregs [2], [19], [20], [27]. On the other hand, apoptotic tumor cells normally stimulate the maturation rather than tolerance of DCs. However, the proportion of Tregs significantly increased in vivo when CD3 antibodies were used [1]. The difference among apoptotic cells may contribute to the different functions of DCapos. Considering the various subtypes of DCs in vivo, recent reports showed that CD8^+^ spleen DCs could secrete TGF-beta and directly induce Treg proliferation, whereas the CD8^–^ DCs could induce Treg proliferation only in the presence of high doses of exogenous TGF-beta. [28] This may explain why bone-marrow-derived myeloid DCapos could not induce Treg proliferation. In our system, intraperitoneal injection might cause apoptotic cell clearance by dendritic cells and macrophages, which is different from the results obtained with intravenous injection. Thus, our results showed that Tregs might not contribute to the immunosuppression of myeloid DCapos.

We put NO at the center of the immunosuppression mediated by apoptotic cells. While NO secretion by DC depends on iNOS, iNOS activation in the DCs after phagocytosing the apoptotic cells remains to be investigated. Previous studies showed that STAT3 is a cell intrinsic negative regulator of DC activity [15], [16], [29], [30]. Inhibition of Janus kinase 2 (Jak2)/STAT3 signaling resulted in dramatic activation of immature DCs including upregulation of MHC class II, co-stimulatory molecules, and a dramatic increase in the ability to stimulate Ag-specific T cells [16]. Sun et al. found that histone deacetylase modulates immune responses depended on the acetylation and activation of STAT3 in DCs, which promoted the transcription of IDO [31]. Knockout of the Jak family member Tyk2 led to selective deficiency in STAT3 activation in respective cytokine receptor signal and Tyk2^–/–^ macrophages fail to produce nitric oxide upon lipopolysaccharide induction [32], [33]. Furthermore, the Jak inhibitor AG490 could markedly inhibit iNOS transcription and NO release in the microglia [34]. Contrasting from the current results, Tassiulas et al. reported that apoptotic cell binding fails to phosphorylate STAT3 in human and murine macrophage [35]. Our results confirmed that co-culture with apoptotic cells could also induce STAT3 phosphorylation in DCs, leading to enhanced NO secretion. Although this result was consistent with the phenomenon observed by most other researchers [33], [34], [36], [37], it does not exclude the possibility that other signaling pathways such as STAT1 may be involved in the generation of NO.

The mechanisms of STAT3 signaling activation in DC after apoptotic cells pahgotosis remain to be elucidated. Some molecules displayed on the surface of apoptotic cells (AC), including phosphatidylserine, are recognized as “eat-me” signals by DCs. Research has indicated that both macrophages and DCs require MerTK binding of apoptotic cells to mediate phagocytosis through phosphatidylserine [14], [38], [39]. Further, apoptotic cells could not convert MerTK^–/–^ DCs to tolerogenic DCs [40]. Although it has been reported that MerTK phosphorylation resulted in c-Src-mediated activation of STAT3 [21], [41], the intermediate(s) between MerTK and STAT3 are yet to be determined. Some studies have reported that MerTK binding of apoptotic cells is an upstream event of PI3K activation [14], [21]. There might be other intermediate(s) between MerTK and PI3K, although it is not clear how PI3K activation triggers STAT3 phosphorylation. Zhou et al. found that mammalian target of rapamycin (mTOR) was downstream of PI3K, and it activated STAT3 in cancer stem-like cells [42]; this mechanism needs to be explored in DCs. On the other hand, studies have reported that inhibition of the mTOR pathway in DCs could increase the secretion of proinflammatory cytokines and the activation of NF-kappaB, whereas STAT3 phosphorylation is blocked [43], [44]. NF-kappaB is a transcription factor that regulates the expression of several genes that control the activation, maturation, and antigen-presenting function in DCs. This result coincides with that of another study in which apoptotic cells were reported to lead to the inhibition of NF-kappaB pathway and the activation of STAT3 [21].

In summary, we demonstrated that myeloid DCapos could directly inhibit the immune reponses via NO which is induced after activation of the STAT3 signaling pathway by phagocytosis activation-induced apoptotic CD4^+^ T cells. These findings shed more insights into the mechanism by which regulatory DCs negatively regulate antigen-specific immune responses. Targeting these molecules may provide an approach to induce or eliminate the tolerogenic DCs in the immunotherapy of autoimmune diseases.
